# Lack of Support for the Association between *GAD2* Polymorphisms and Severe Human Obesity

**DOI:** 10.1371/journal.pbio.0030315

**Published:** 2005-08-30

**Authors:** Michael M Swarbrick, Björn Waldenmaier, Len A Pennacchio, Denise L Lind, Martha M Cavazos, Frank Geller, Raphael Merriman, Anna Ustaszewska, Mary Malloy, André Scherag, Wen-Chi Hsueh, Winfried Rief, Franck Mauvais-Jarvis, Clive R Pullinger, John P Kane, Robert Dent, Ruth McPherson, Pui-Yan Kwok, Anke Hinney, Johannes Hebebrand, Christian Vaisse

**Affiliations:** **1**Diabetes Center, University of California, San Francisco, California, United States of America,; **2**Department of Child and Adolescent Psychiatry, University of Duisburg-Essen, Essen, Germany,; **3**Department of Energy Joint Genome Institute, Walnut Creek, California, United States of America,; **4**Cardiovascular Research Institute, University of California, San Francisco, California, United States of America,; **5**Institute of Medical Biometry and Epidemiology, Phillips-University of Marburg, Marburg, Germany,; **6**Department of Medicine, University of California, San Francisco, California, United States of America,; **7**Department of Psychology, University of Marburg, Marburg, Germany,; **8**Division of Diabetes, Endocrinology and Metabolism, Baylor College of Medicine, Houston, Texas, United States of America,; **9**Ottawa Health Research Institute, Ottawa, Ontario, Canada,; **10**University of Ottawa Heart Institute, Ottawa, Ontario, Canada; University of OxfordUnited Kingdom

## Abstract

The demonstration of association between common genetic variants and chronic human diseases such as obesity could have profound implications for the prediction, prevention, and treatment of these conditions. Unequivocal proof of such an association, however, requires independent replication of initial positive findings. Recently, three (−243 A>G, +61450 C>A, and +83897 T>A) single nucleotide polymorphisms (SNPs) within glutamate decarboxylase 2 *(GAD2)* were found to be associated with class III obesity (body mass index > 40 kg/m^2^). The association was observed among 188 families (612 individuals) segregating the condition, and a case-control study of 575 cases and 646 lean controls. Functional data supporting a pathophysiological role for one of the SNPs (−243 A>G) were also presented. The gene *GAD2* encodes the 65-kDa subunit of glutamic acid decarboxylase—GAD65. In the present study, we attempted to replicate this association in larger groups of individuals, and to extend the functional studies of the −243 A>G SNP. Among 2,359 individuals comprising 693 German nuclear families with severe, early-onset obesity, we found no evidence for a relationship between the three *GAD2* SNPs and obesity, whether SNPs were studied individually or as haplotypes. In two independent case-control studies (a total of 680 class III obesity cases and 1,186 lean controls), there was no significant relationship between the −243 A>G SNP and obesity (OR = 0.99, 95% CI 0.83–1.18, *p* = 0.89) in the pooled sample. These negative findings were recapitulated in a meta-analysis, incorporating all published data for the association between the −243G allele and class III obesity, which yielded an OR of 1.11 (95% CI 0.90–1.36, *p* = 0.28) in a total sample of 1,252 class III obese cases and 1,800 lean controls. Moreover, analysis of common haplotypes encompassing the *GAD2* locus revealed no association with severe obesity in families with the condition. We also obtained functional data for the −243 A>G SNP that does not support a pathophysiological role for this variant in obesity. Potential confounding variables in association studies involving common variants and complex diseases (low power to detect modest genetic effects, overinterpretation of marginal data, population stratification, and biological plausibility) are also discussed in the context of *GAD2* and severe obesity.

## Abstract 2

A large genetic study involving multiple populations is not able to replicate previous findings linking variation in the *GAD2* gene to susceptibility to obesity.

## Introduction

By dramatically increasing mortality [1] and morbidity [2] from cardiovascular disease, obesity has emerged as a major public health issue for the 21st century. Obesity is strongly associated with type 2 diabetes, hypertension, dyslipidemia, heart failure, and stroke [3]. This burden of disease is particularly high in individuals with class III obesity (body mass index [BMI] > 40 kg/m^2^), as they are more likely to develop at least one of these co-morbidities [4].

The importance of genetic factors in determining susceptibility to obesity has been well established elsewhere, by studies of twins [5], and adoptees [6]. At present, there is support for a model in which the propensity to become obese is determined largely by genetic factors, with environmental factors determining the expression of the condition [7]. These genetic influences are likely to be particularly powerful in individuals with severe or early-onset forms of obesity [8]. While several rare monogenic forms of non-syndromic obesity have been described to date [9–13], efforts aimed at identifying common susceptibility alleles for the condition have been much less successful [14].

The Chromosome 10p12 region has previously demonstrated significant linkage with severe human obesity [15]. In the initial study [15] involving individuals ascertained by a proband with class III obesity (BMI > 40 kg/m^2^) and at least one sibling with BMI > 27 kg/m^2^, strong evidence for linkage (maximum logarithm of odds score 4.85) was obtained at the marker D10S197. The linkage peak encompassed a region of approximately 15 centimorgans. Confirmation of this linkage, albeit at lower levels of significance, was obtained in German Caucasians [16] and a combined sample of Caucasian Americans and African Americans [17]. The marker D10S197 is located within intron 7 of the glutamate decarboxylase 2 *(GAD2)* gene, which encodes the 65-kDa subunit of glutamic acid decarboxylase—GAD65.

Recently, Boutin et al. [18] obtained evidence to implicate *GAD2* as a candidate gene for human obesity. In a case-control study for class III obesity, the authors identified both a haplotype (consisting of the most frequent alleles of single nucleotide polymorphisms +61450 C>A and +83897 T>A), and a SNP (−243 A>G) within *GAD2* that differed in frequency between cases and controls. In family-based tests of association involving 612 individuals from 188 nuclear families, the +61450 C>A and +83897 T>A SNPs were associated with class III obesity. The “protective” wild-type (WT) haplotype (+61450 C and +83897 T) identified in the case-control study was found to be in excess in unaffected offspring.

As the *GAD2* variant allele −243 G was in the 5′ region of the gene (the other two SNPs were located in intronic regions), displayed the strongest association with class III obesity in the case-control study, and was in linkage disequilibrium with the +61450 C>A and +83897 T>A SNPs, functional studies were performed to test its effects on transcription and nuclear protein binding. In a luciferase reporter gene containing the *GAD2* promoter, the −243 G allele increased the transcriptional activity 6-fold relative to an equivalent reporter gene containing the WT (−243 A) allele in βTC3 murine insulinoma cells. Also, relative to the WT (A) allele, oligonucleotide probes containing the variant (G) allele had a higher affinity for an unidentified nuclear protein from βTC3 cells. Overall, their results suggested that the *GAD2* −243 G allele might not only constitute a genetic marker for class III human obesity, but may also exert a significant physiological effect.

In recent years, many unreplicated associations have been reported between common genetic polymorphisms and measures of adiposity [19,20]. Indeed, one of the significant challenges in genetic association studies is the presence of statistical trends towards susceptibility (with the suspected allele being neither sufficient nor necessary for disease expression), rather than clear cause-and-effect relationships. This factor reduces the power of individual studies; consequently, it has become critical to develop larger multi-center studies to confirm positive associations in other populations, and to perform meta-analyses to more accurately estimate the magnitude of the genetic effect [21]. In the present study, we attempted to replicate the recent findings of Boutin et al. [18] by performing family-based tests of association and case-control studies in three Caucasian populations.

## Results

### Family-Based Tests of Association

In the previous report [18], an excess of WT alleles was observed in unaffected offspring for the +61450 C>A and +83897 T>A SNPs (*p* = 0.03 for each), and the haplotype consisting of the WT alleles at these SNPs was found to be in excess in unaffected offspring (*p* = 0.05). However, excess transmission of the G allele of the −243 A>G SNP to affected offspring was not observed (*p* = 0.06).

To further assess these initial findings in a much larger cohort, we performed family-based tests of association in 2,359 German Caucasian individuals from 693 nuclear families. Nuclear families were composed of obese children (mean BMI percentile = 98.6 ± 2.3, range 90th–100th percentile), their obese siblings, and both of their parents. The clinical characteristics of the nuclear families segregating obesity are shown in Table 1. This group of individuals included the 89 families that had previously displayed suggestive linkage for obesity (maximum likelihood binomial logarithm of odds score of 2.24) at D10S197 [22].

**Table 1:**
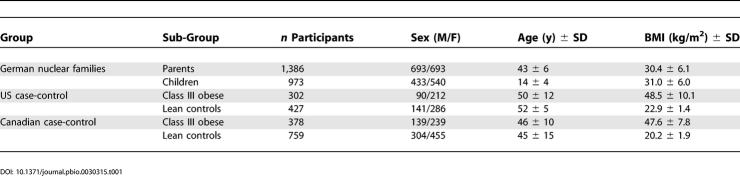
Clinical Characteristics of Participants Used for Family-Based Association and Case-Control Studies

Using the pedigree disequilibrium test (PDT) [23], we found no evidence for excess transmission of any *GAD2* alleles to obese children in the 89 families displaying prior linkage of obesity to Chromosome 10p (Table 2). This finding alone suggested that the original linkage signal in this region might be due to different SNPs than the ones under study. We next included samples that were not previously tested for linkage, bringing the total group to 693 nuclear families. As in the linked families, we found no association between any of the *GAD2* SNPs and obesity (Table 2). Similarly, studies of haplotype transmission in the entire group using the transmission disequilibrium test (TDT) did not provide any evidence for a “protective” haplotype consisting of the +61450 C and +83897 T alleles, or any other two- or three-allele *GAD2* haplotypes (Table 3).

**Table 2:**
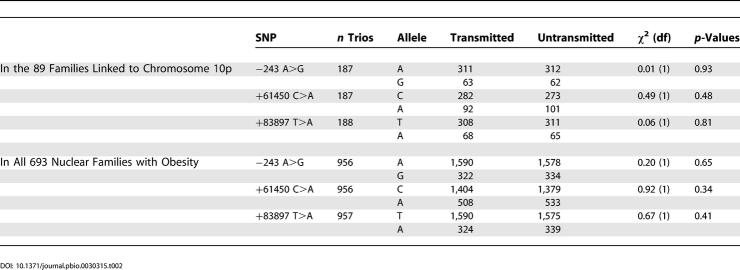
PDT Results for *GAD2* SNPs in 693 German Families Segregating Severe, Early-Onset Obesity

**Table 3:**
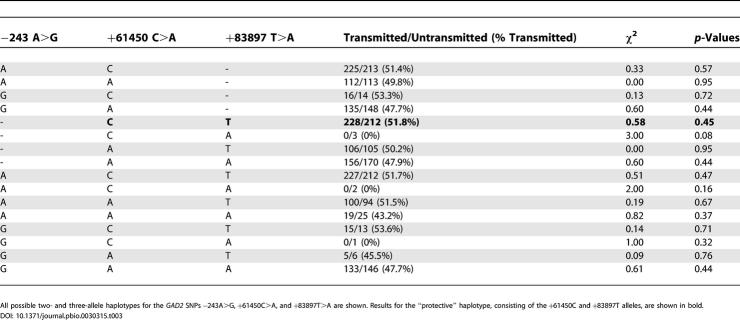
Analysis of Haplotype Transmission in German Obesity Trios Using the TDT

### Case-Control Studies

Previously [18], the −243 A>G, +61450 C>A, and +83897 T>A SNPs were associated with class III obesity in one case-control group (349 obese cases and 376 nonobese controls), whereas the association between the −243 A>G SNP and obesity was significant in the pooled sample of 575 obese cases and 646 controls (odds ratio [OR] of 1.3, 95% confidence interval [CI] 1.053–1.585, *p* = 0.014). We attempted to replicate these associations by performing case-control studies of class III obesity in two groups of North American Caucasians, one from the United States and the other from Canada. The clinical characteristics of the participants used in the case-control studies are shown in Table 1.

#### US case-control study

Each of the three *GAD2* polymorphisms (−243 A>G, +61450 C>A, and +83897 T>A) was found to be in Hardy-Weinberg equilibrium in 302 class III obese (BMI > 40 kg/m^2^) cases and 427 lean controls (Table 4). None of the three variant alleles were associated with class III obesity (−243 G allele and class III obesity [OR = 1.11, 95% CI 0.84–1.46, *p* = 0.45]; the +61450 A allele [OR = 1.25, 95% CI 0.99–1.57, *p* = 0.058]; the +83897 A allele [OR = 1.14, 95% CI 0.87–1.50, *p* = 0.33]). Moreover, within the obese and lean groups, there was no association between *GAD2* genotype and BMI for any of the three SNPs studied (unpublished data).

**Table 4:**
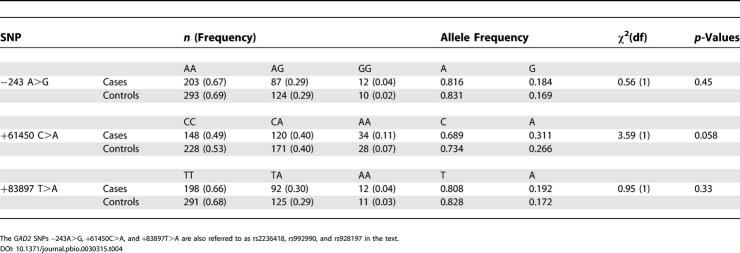
Genotype Results for US Case-Control Study

#### Canadian case-control study

The Canadian participants were also genotyped for the *GAD2* −243 A>G, +61450 C>A, and +83897 T>A SNPs. Genotypes of both cases and controls conformed to Hardy-Weinberg equilibrium, and the allele frequencies were similar to those observed in US Caucasians (Table 5).

**Table 5:**
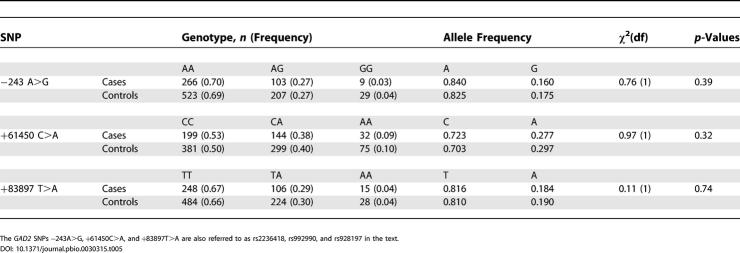
Genotype Results for Canadian Case-Control Study

As for US participants, the frequency of the −243 G allele did not differ between a group of 378 class III obese Canadian participants (frequency = 0.160) and a group of 759 lean controls (frequency = 0.175). The −243 G allele was not associated with class III obesity in this case-control study (OR = 0.90, 95% CI 0.71–1.14, *p* = 0.39). Pooling the results from the US and Canadian studies (680 class III obese cases and 1,186 lean controls) did not provide significant evidence for an association between the −243 G allele and class III obesity (OR = 0.99, 95% CI 0.83–1.18, *p* = 0.89).

#### Meta-analysis for the −243 A>G variant

**Figure 1:**
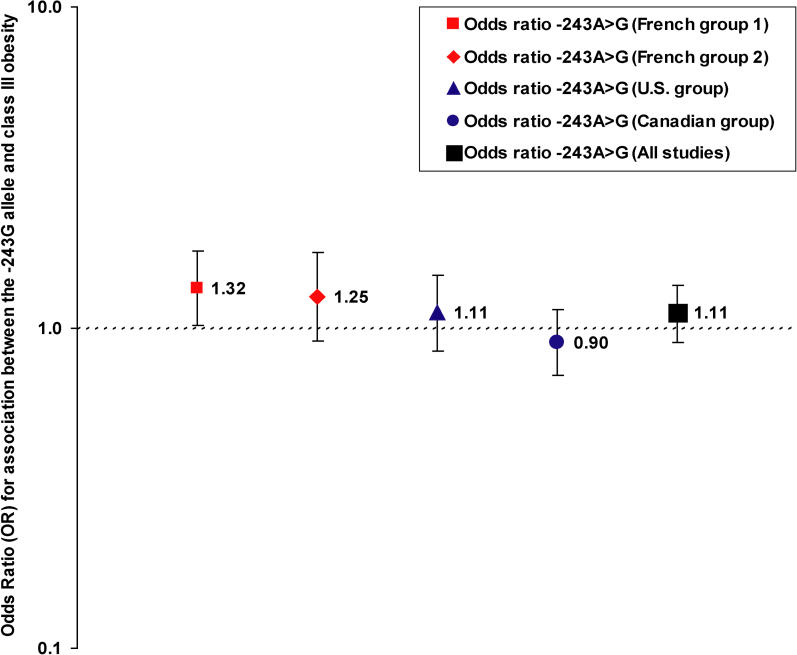
Meta-Analysis for the Association between the *GAD2* −243 A>G Polymorphism and Class III Obesity French groups 1 and 2 refer to the genotype results from two sets of Caucasian class III obese cases and controls studied by Boutin et al*.* [18]. US and Canadian groups were from the present study. The meta-analysis for the association between the −243 G allele and class III obesity yielded a summary OR of 1.11 (95% CI 0.90–1.36), obtained in a total sample of 1,252 class III obese cases and 1,800 controls using a Mantel-Haenszel method and a fixed effects model.

It has been proposed elsewhere [24] that the interpretation of results from association studies may be aided by meta-analysis of all similar studies. We compiled all available genotyping data (ours and that of the previous *GAD2* study [18]) pertaining to the relationship between the −243 A>G polymorphism and class III obesity, and performed a meta-analysis (Figure 1). Inclusion of the data from the original study and our two case-control studies (a total of 1,252 cases and 1,800 controls) yielded an OR of 1.11 (95% CI 0.90–1.36) for the association between the −243 G allele and class III obesity.

### Further Investigation of *GAD2* as a Candidate Gene for Severe Obesity

In order to evaluate the potential relationship between other common SNPs in *GAD2* and severe obesity, we conducted a comprehensive investigation of haplotype structure in this region using the data from the International HapMap Project (http://www.hapmap.org/cgi-perl/gbrowse/gbrowse/hapmap) and Haploview (http://www.broad.mit.edu/mpg/haploview/index.php) [25]. As described earlier by Boutin et al., *GAD2* (and 2 kilobases of its promoter) lies within a 90-kilobase block of linkage disequilibrium on Chromosome 10p12 (Figure S1). Two of the three SNPs used in this study, −243 A>G (rs2236418) and +83897 T>A (rs928197), were used in the construction of HapMap. To incorporate the data from the third SNP, +61450 C>A (rs992990), into this framework, we genotyped the same CEPH (Centre D'étude du Polymorphisme Humain) samples (Utah residents with ancestry from northern and western Europe) that were used in the creation of the map. When the genotype results from the +61450 C>A SNP were integrated with those from HapMap, the overall structure of the haplotype block did not change. To capture at least 95% of the haplotype diversity within this haplotype block, we then determined that genotyping of three more SNPs was required: rs3781117 (intron 4), rs3781118 (intron 4), and rs1330581 (intron 7) (Figure S2).

The German families and case-control participants from the US and Canada were genotyped for the SNPs rs3781117, rs3781118, and rs1330581. None of these three SNPs were transmitted to affected children more frequently than expected by chance (Table S1). Inclusion of these *GAD2* SNPs with the original three did not yield significant results for association between any *GAD2* haplotype and obesity (Table 6). Moreover, rs3781117, rs3781118, and rs1330581 were not independently associated with class III obesity in either the US or Canadian case-control study groups (Table S2).

**Table 6:**
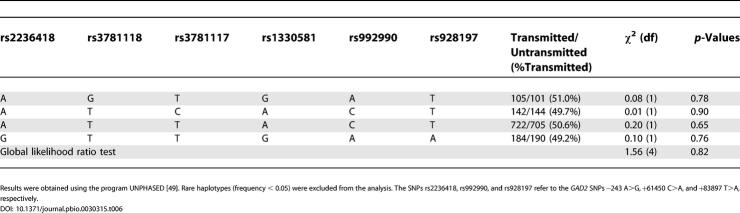
TDT Results for Six SNPs Spanning the GAD2 Haplotype Block

#### Ethnic differences in *GAD2* allele frequency

The presence of an underlying population substructure, resulting from ethnic admixture, is a common bias in association studies [26]. We were therefore interested in determining whether different ethnic groups could display significant differences in *GAD2* allele frequency.

In samples obtained from the Human Variation Collection (Coriell Institute for Medical Research, Camden, New Jersey, United States), the frequencies of *GAD2* alleles in Caucasians were comparable with those observed for the Caucasian groups tested in previous studies (Table S3). However, we observed marked and highly significant differences in allele frequency for the −243 A>G SNP between Caucasians and populations of West African origin represented by samples collected in the US or in France. North African populations presented an intermediate allelic distribution.

### Reporter Gene Assay for *GAD2* −243 G Promoter Variant

In addition to the aforementioned genetic results, Boutin et al. [18] also found that luciferase reporter genes containing the −243 G allele in the *GAD2* promoter (from −1710 to −4, relative to the transcriptional start site) displayed a 6-fold higher activity compared to reporter genes containing the −243A allele in βTC3 murine insulinoma cells (Figure 3 in [18]). We were interested in investigating the nature of this allele-specific difference in *GAD2* promoter activity, with the goal of identifying the specific *cis*-acting elements responsible. To accomplish this, we also tested the effect of the −243 A>G SNP on transcription of a luciferase reporter gene in βTC3 cells.

We found that introduction of the −243 G allele into the −1710/−4 reporter gene did not elicit detectable effects on luciferase transcription relative to the WT reporter gene (Figure S3). Similarly, we could not detect any allele-specific effects of the −243 A>G polymorphism in two smaller reporter genes containing the *GAD2* promoter (from −501 to −4 and from −1,234 to −4). However, the transcriptional activity of our WT −1710/−4 reporter gene was appreciably higher than that of pGL3Basic in βTC3 cells, suggesting that the *GAD2* promoter does exhibit some basal transcriptional activity in this cell line.

### Electrophoretic Mobility Shift Assay

In the previous study [18], oligonucleotide probes containing either of the −243 A>G alleles were tested for their affinity for nuclear extract prepared from βTC3 murine insulinoma cells. The probe containing the −243 G allele was found to have a 6-fold higher affinity for an unidentified nuclear protein (Figure 4 in [18]). However, the DNA–protein complex was also present to some extent in the negative control lanes (lacking nuclear extract), and the oligonucleotide probes differed with respect to their specific activity.

We obtained the sequences of oligonucleotide probes used by the authors, and utilized the electrophoretic mobility shift assay (EMSA) to confirm this allele-specific difference in binding affinity. We were also interested in determining whether this effect was specific for neuronal or β cells relative to other cell lines. Our experiments indicated that the −243 A allele had a greater affinity for an unidentified protein from βTC3 nuclear extracts relative to the −243 G allele (Figures S4 and S5). Our results were not consistent with those previously described by Boutin et al. [18].

## Discussion

In the present study, we attempted to replicate the important recent findings of Boutin et al. [18], which implicated three SNPs in *GAD2* (the −243 A>G allele and a haplotype of the +61450 C>A and +83897 T>A SNPs) in the predisposition to class III human obesity. To replicate their findings, we first performed family-based tests of association for all three SNPs in 693 nuclear families segregating severe obesity (2,359 participants, nearly four times as many participants as in the original report). This group of individuals included 89 families found to have linkage of severe obesity to Chromosome 10p12 [16,22]. No evidence for excess transmission of any *GAD2* alleles or haplotypes from parents to affected offspring was obtained. Next, we conducted an adequately powered case-control study to test the association between class III obesity and the *GAD2* −243 A>G variant in Caucasians. Consistent with the family-based association results, we did not observe any association between the −243 G variant and class III obesity in 680 cases and 1,186 lean controls. These findings were also obtained in a meta-analysis for the association between the −243 A>G SNP and class III obesity. Lastly, we obtained results from the reporter gene and DNA binding experiments for the −243 A>G variant that were inconsistent with the original report. Overall, we found that (i) a haplotype consisting of the WT alleles at SNPs +61450 C>A and +83897 T>A does not appear to protect against severe, early-onset obesity, (ii) the −243 A>G SNP is not associated with class III obesity in adults, (iii) other haplotypes in the region of *GAD2* are not associated with severe obesity, and (iv) the −243 A>G SNP does not elicit detectable effects on transcription of a luciferase reporter gene in βTC3 murine insulinoma cells.

Irreproducibility of positive findings has been a common criticism leveled at association studies investigating the common genetic basis of complex diseases [19,24]. The reasons cited are numerous, and include a lack of statistical power to detect small to moderate effects, lack of control over the Type I error rate, overinterpretation of marginal data, population stratification, and poor biological plausibility [27,28]. Regarding the conflicting results obtained by Boutin et al. [18] and the current study, it is likely that the lack of replication could be ascribed to any of these causes, which are discussed below. The inconsistencies between association studies may also reflect the complex interactions between multiple population-specific genetic and environmental factors.

The lack of statistical power to detect alleles of minor effect is likely to have contributed to the differences between the study by Boutin et al. [18] and the current investigation. Based on the findings of the initial report, we conducted an adequately powered, ethnically matched, case-control study. Although our results overlapped with the size of the initial effect, they did not show a significant association between the −243 G allele and class III obesity (Figure 1). We estimate that we had 60% power to detect a significant difference (α of 0.05) in allele frequency between our pooled groups of cases and controls, assuming that the −243 G allele (frequency of 0.18) was the disease allele, a genotype relative risk of 1.25, and a prevalence of class III obesity in the general population of 5% [29]. The family-based association tests had a similar amount of power (∼60%), given the same assumptions. Under these conditions, the original study [18] may have been underpowered. Moreover, it must be pointed out that the marginally significant association (*p* = 0.04) they observed between the −243 G allele and class III obesity was observed in only one of their two groups of participants, and did not reach nominal significance in their family-based analysis (*p* = 0.06). Although the lack of statistical significance does not exclude the possibility of an association (as we cannot rule out smaller effects), the data do not support a relationship between this SNP and class III obesity.

The interpretation of results from genetic association studies is frequently complicated by other statistical issues, such as a failure to control for multiple hypothesis testing, overinterpretation of marginal data as positive trends, and the well-documented tendency for initial positive findings to overestimate the strength of the association [21]. This “jackpot” phenomenon [24] can be readily observed in our meta-analysis (Figure 1).

Population stratification may also account for some of the inconsistencies observed between association studies, though its importance may have been overestimated [19,26]. Population stratification is usually controlled for by careful matching of cases and controls by ethnicity, using family-based tests of association (such as the TDT) or studying multiple case-control populations [30]. Considering the marked differences in allele frequency that we observed between ethnic groups for the *GAD2* SNPs (the −243 A>G and +61450 C>A SNPs in particular), as well as the known differences in the prevalence of class III obesity between Caucasian Americans and African Americans [31], it is plausible that a small difference in ancestry between cases and controls could lead to spurious claims of association. Naturally, future studies of the *GAD2* gene should carefully take this into consideration.

There is no obvious explanation for the differences in results obtained for the EMSA and reporter gene assays. Regarding the EMSA, a major problem with these experiments is that most random DNA sequences will be bound by a nuclear extract from any cell line (Figures S4 and S5 and Figure 4 in [18]). It is likely that the introduction of single base-pair differences into this DNA sequence will interfere with the binding pattern observed. Moreover, while an allele-specific difference in the binding of βTC3 cell nuclear extract definitely occurs for the −243 A>G polymorphism, this observation is of limited physiological significance, because: (i) it appears to be restricted to this cell type (and there is no apparent difference in allele-specific binding for nuclear extract derived from a neuronal cell line); and (ii) the binding of this nuclear protein does not appear to affect transcription of a luciferase reporter gene in βTC3 cells. Finally, even if the −243 A>G SNP did affect transcription of the reporter gene in this context, there is no prior biological evidence to suggest that perturbation of *GAD2* expression in β cells could exert detectable effects on long-term energy homeostasis.

This latest point raises the issue of biological plausibility. *GAD2* encodes the 65-kDa isoform of the enzyme glutamate decarboxylase, which catalyzes the production of γ-aminobutyric acid (GABA), a major inhibitory neurotransmitter, from glutamic acid. The biological evidence implicating *GAD2* as a candidate gene (and by extension, hypothalamic GABA levels as causative) in severe obesity is as follows: *GAD2* mRNA is co-expressed with neuropeptide Y in neurons of the hypothalamic arcuate nucleus that act in the nearby paraventricular nucleus and other hypothalamic areas to stimulate food intake [32]. Concomitantly, these arcuate neuropeptide Y neurons inhibit the parallel and opposing effects of neighboring pro-opiomelanocortin/cocaine- and amphetamine-regulated transcript neurons via GABA-ergic collateral inputs [33]. In rats, administration of muscimol, a GABA_A_ receptor agonist, into either the third ventricle or the hypothalamic paraventricular nucleus stimulates feeding in a dose-dependent manner [34]. Similarly, inhibition of GABA synthesis in the ventromedial hypothalamus, by injection of antisense GAD-65 and GAD-67 oligonucleotides, has been shown to suppress food intake [35].

However, enthusiasm for *GAD2* as a candidate gene for severe obesity is dampened somewhat by the observation that *GAD2*-deficient mice appear normal with respect to behavior, locomotion, reproduction, and glucose homeostasis, but suffer from epileptic seizures [36]. Also, levels of *GAD2* mRNA in the arcuate nucleus of the rat do not change in response to 48 hr of food deprivation, as do levels of prepro–neuropeptide Y mRNA [37]. Furthermore, the notion that hypothalamic GABA levels are proportional to food intake may be an oversimplification; although microinjection of GABA into the paraventricular nucleus and ventromedial hypothalamus stimulates feeding [38], injection of GABA, muscimol [39], or an adenovirus expressing *GAD2* [40], into the lateral hypothalamus of rats has been observed to have the opposite effect.

While these experimental results do not exclude *GAD2* as a candidate gene for human obesity, it remains possible that the linkage signal could be due to variation in a neighboring gene. Certainly *GAD2* is the leading candidate in this region, due to some of the biological evidence presented above and the location of D10S197 within one of its introns. However, in light of the large number of genes involved in energy homeostasis (recently reviewed in [41] and [42]), the multiple tissue-specific roles of each gene, and the readily available information regarding the homology and expression pattern of uncharacterized genes, it now seems possible to make a tenuous case for almost any single gene in the regulation of body weight. For example, a preliminary glance at the Chromosome 10p12 region yielded several interesting genes: *TPRT* (trans-prenyltransferase), the enzyme that elongates the prenyl side-chain of coenzyme Q, one of the key elements of the respiratory chain within mitochondria; *GPR158*, which encodes a metabotropic glutamate, GABA_B_–like G-protein-coupled receptor; and *PTF1A,* which encodes pancreas-specific transcription factor 1a. Although only a little is known about each of these genes, it is possible to speculate on the potential role of each in obesity. *GAD2* is no exception. At present, however, there is insufficient genetic or biological evidence to implicate genetic variation in *GAD2* in the predisposition to severe obesity in humans.

## Materials and Methods

### Participants

The ascertainment strategy for these participants has been described previously [43]. BMI was calculated as weight in kg/(height in m)^2^. For the PDT and TDT analyses, we genotyped 973 (extremely) obese children and adolescents (693 probands and 280 of their siblings: mean age 14.0 ± 3.7 y, mean BMI 31.0 ± 6.0 kg/m^2^) and both of their parents (mean age 42.7 ± 5.9 y, mean BMI 30.4 ± 6.1 kg/m^2^). Written informed consent was given by all participants and, in the case of minors, their parents. The Ethics Committee of the University of Marburg approved the study.

Participants were selected from the Cardiovascular Research Institute Genomic Resource in Arteriosclerosis, a population-based investigation of dyslipidemia and atherosclerotic heart disease established at the University of California, San Francisco (UCSF) in California, United States. This population includes patients from the Lipid Clinic of UCSF [44,45], from the UCSF Interventional Cardiology Service, and from collaborating cardiology clinics throughout California. The UCSF Committee on Human Research approved the protocols, and informed written consent was obtained from all patients. From this study group, we selected class III obese (BMI ≥ 40 kg/m^2^) non-Hispanic Caucasian individuals, as well as a group of lean individuals (mean BMI = 22.9 kg/m^2^, range 20.0–25.6 kg/m^2^) with a similar age and sex distribution. Genomic DNA was extracted from buffy coats using a Puregene DNA Purification Kit from Gentra Systems (Minneapolis, Minnesota, United States).

Obese Caucasian individuals with a mean BMI of 48 kg/m^2^ (range 36–81 kg/m^2^, with 91% of individuals having a BMI > 40 kg/m^2^) were recruited from the Ottawa Hospital Weight Management Clinic. Age- and sex-matched lean Caucasian individuals with a BMI below the 10th percentile for age and sex were recruited as controls from the Ottawa region. The Human Ethics Research Boards of the Ottawa Hospital and the University of Ottawa Heart Institute approved the study. Informed written consent was obtained from all participants.

### Genotyping

Three *GAD2* SNPs were genotyped by PCR-based restriction fragment length polymorphism analysis or by tetra-amplification refractory mutation system PCR. To detect rs2236418 (−243 A>G), a PCR-amplicon of 636 base pairs (bp) (primers: GAD2–243-F: 5′- GGAGCCAGACCTCAAACAAA-3′ and GAD2–243-R: 5′- TTTGGAGACTGGAGCAGGTC-3′) was digested by DraI ([NEB GmbH, Frankfurt am Main, Germany]; 2 h at 37 °C; A-allele: 395 bp and 241 bp; G-allele: undigested). To detect rs928197 (+83897 T>A), a PCR-amplicon of 242 bp (primers: GAD2–83897-F: 5′- GTGGCAGGCAGCTGATAGTC-3′ and GAD2–83897-R: 5′- CACCTGTGGGACAGACCATA-3′), was digested by AluI ([Fermentas GmbH, St. Leon Rot, Germany]; 2 h at 37 °C; T-allele: 146 bp and 96 bp; A-allele: undigested). PCR products of all SNPs were electrophoresed in 2.5% agarose gels stained with ethidium bromide.

SNP rs992990 (+61450 C>A) was genotyped by tetra-amplification refractory mutation system PCR [46]. Primers were as follows: GAD-61450-FiC 5′- ATTCTTACTGACAAAGCTGAGTTTACCC-3′ and GAD-61450-Ro 5′- TATTTAGGTGAAGTGCTTAGAACTGTGC-3′ 199-bp amplicon detects the C-allele; GAD-61450-RiA 5′- TCATGTTCTATGGCTAGATGTCTAATCCT-3′ and GAD-61450-Fo 5′- GGCAGCTTCTCTTCTAAAAAGACAAATA-3′ 151-bp amplicon detects the A-allele. The amplicon length of the two outer primers (GAD-61450-Fo and GAD-61450-Ro) was 294 bp.

Positive controls of variant genotypes were run on each gel. To test validity of the genotypes, allele determinations were rated independently by at least two experienced persons. Discrepancies were resolved unambiguously, either by reaching consensus or by retyping.

Genotyping of the US participants for the *GAD2* SNPs rs2236418, rs992990, and rs928197 was performed using fluorescently labeled allele-specific primer extension, assayed by fluorescence polarization template-directed dye incorporation [47]. The primers used to amplify the region around each SNP were as follows: rs2236418p1 CCTCCCTCTCTCGTGTTTTT, rs2236418p2 GTGTCACGCAGGAACAGAAA, rs928197p1 CCTCTTATCACTTGCAGGATCT, rs928197p2 GTGGTTCCATACTCCATCATTC, rs992990p1 GGGACAGAGAATTCAGTGACAG, and rs992990p2 GTCATTTGTGAGCTTGGTGAC. Single-base extension reactions for each SNP were performed using the primers rs2236418p4 TTGGAAGCCGGGGAGC, rs928197p3 AAACAATAAGGTTCTGACTGTTGAGC, and rs992990p4 CATGTTCTATGGCTAGATGTCTAATTC.

To test for ethnicity-specific differences in allele frequency, we also genotyped 99 Caucasian-American and 99 African-American individuals from the Human Variation Collection (Coriell Institute for Medical Research, Camden, New Jersey, United States), as well as 60 West Africans and 36 North Africans living in Paris, France.

To capture more than 95% of the haplotype diversity in the *GAD2* region, we also genotyped the SNPs rs3781117, rs3781118, and rs1330581 (Tables 6, S1, and S2) using fluorescence polarization template-directed dye incorporation. The primers used were as follows: rs3781117p1, rs3781117p2, rs3781118p1, rs3781118p2, rs1330581p1, and rs1330581p2 (sequences available from the authors on request). For each of these SNPs, single-base extension reactions were performed with the primers rs3781117p3, rs3781118p3, and rs1330581p3 (as above).

Genotyping of the Canadian participants for the −243 A>G polymorphism was performed by PCR amplification with the primers GAD2_243A>G_ALU_F ( GGCTCCCTTTCCCTCAAAT) and GAD2_243A>G_ALU_R ( ATAACGTGTGTGTATGCGAGCTGGAGA) followed by digestion with AluI. When separated by agarose gel electrophoresis, this produced a unique set of bands corresponding to each genotype: AA (20, 47, and 92 bp), AG (20, 47, 92, and 139 bp), and GG (20 and 139bp).

For the SNPs (−243 A>G, +61450 C>A, and +83897 T>A), 94 DNA samples from each laboratory (Marburg, Germany; and San Francisco, California, United States) were exchanged and genotyped according to the other laboratory's method. Three discrepancies (out of 564 genotypes) were observed, resulting in a between-laboratory error rate of < 1%.

Family-based tests of association for single markers were carried out using the PDT, which accounts for the dependency between the sibs [23]. Haplotype TDTs for two and three markers were performed using the program GeneHunter, version 2.0 beta [48] (available at http://helix.nih.gov/apps/bioinfo/genehunter.html). Here, transmissions were counted only when phase could be determined unambiguously. TDT analysis of all six *GAD2* SNPs (comprising the haplotype block) was performed using the program UNPHASED [49] implementing the EM (expectation-maximization) algorithm (available at http://www.mrc-bsu.cam.ac.uk/personal/frank/). For the US and Canadian participants, comparisons between cases and controls for allele frequency were performed using a two-tailed χ^2^ test, and *p*-values were calculated using the program GraphPad Prism version 3.0a for Macintosh (GraphPad Software, San Diego California, United States). For the meta-analysis, we used the Mantel-Haenszel method to calculate stratified summary effects using a fixed effect model. Power calculations were performed using the Genetic Power Calculator [50] provided at http://statgen.iop.kcl.ac.uk/gpc/.

### Reporter gene constructs

The effects of the −243 A>G polymorphism on *GAD2* transcriptional activity were tested in the context of a luciferase reporter gene. The oligonucleotides used to amplify a 2,200-bp fragment of the *GAD2* promoter (sense- CGGGTCTCTGCTTTGTTAGC and antisense- TTTGGAGACTGGAGCAGGTC) were incorrectly specified in the original report [18], as sequence comparisons between them and the *GAD2* promoter sequence suggested that they would have yielded a 1,706-bp PCR product. Moreover, neither oligonucleotide contained restriction sites for BglII or HindIII, as stated.

After communication with the author, P. Boutin, we amplified the −1710/−4 region using the primers GAD2PROMF3 (our designation) CGGGGTACCCGGGTCTCTGCTTTGTTAGC and GAD2PROMR3 CAAGCTTTGGAGACTGGAGCAGG, digested the PCR product with KpnI and HindIII, and inserted it into the KpnI and HindIII sites in front of the firefly luciferase coding sequence, contained in the vector pGL3Basic (Promega, Madison, Wisconsin, United States). This vector was referred to hereafter as the *GAD2* −1710/−4 construct (numbers refer to the regions of the *GAD2* promoter, relative to the transcriptional start site). The −243 G variant allele was introduced into this construct by PCR amplification of the above fragment from a homozygous patient, digestion of this PCR product with NotI and HindIII, and substitution of this fragment into the NotI-HindIII sites of the WT construct. PCR was performed using TaKaRa LA Taq according to the manufacturer's instructions (TaKaRa Biomedicals, Otsu, Shiga, Japan). Using restriction enzyme digestion of the WT and variant *GAD2* −1710/−4 constructs, we also made shorter *GAD2* promoter reporter genes, referred to as −501/−4 and −1234/−4. All reporter genes were sequenced prior to transfection, and corresponded exactly with the human Chromosome 10 sequence provided on the UCSC Genome Bioinformatics site (http://genome.ucsc.edu).

### EMSA

Sequences for EMSA probes were obtained from P. Boutin. The oligonucleotides used included GAD2A−243AF ( CTCTTTTAA**A** GCTCCCCGGCTTCC), GAD2A−243AR ( GGAAGCCGGGGAGC**T** TTAAAAGAG), GAD2−243GF ( CTCTTTTAA**G** GCTCCCCGGCTTCC**C** TTAAAAGAG). Bases in bold type indicate the differences introduced to reflect the −243 A>G polymorphism. Five hundred nanograms of each forward (F) oligonucleotide were end-labeled with γ-^32^P ATP (Perkin-Elmer, Boston, Massachusetts, United States) using T4 polynucleotide kinase (Promega) at 37 °C for 30 min. Subsequently, 1.5 μg of the corresponding unlabelled reverse (R) oligonucleotide and 50 μl of annealing buffer (100 mM NaCl in TE buffer) were added to each labeled (F) oligonucleotide, and the mixture was incubated for 10 min at 95 °C before being cooled slowly for 1–2 h. The resulting labeled, double-stranded probe was then column-purified (Stratagene NucTrap, La Jolla, California, United States), and the concentration of the probe in the eluate was approximately 10 ng/μl. Unlabeled double-stranded probes for competition experiments were also prepared in a similar manner.

All EMSA experiments were performed in a 20-μl reaction volume containing binding buffer (10 mM HEPES [pH 7.9], 75 mM KCl, 2.5 mM MgCl_2_, 0.1 mM EDTA, 1 mM DTT, 3% Ficoll), polydI/dC (final concentration 50 mg/l in TE), 1 μl nuclear extract, and approximately 3 ng of labeled probe. Nuclear extracts from βTC3, Neuro2A, T98G, HepG2, and HEK293 cells were prepared using the method of Schreiber et al. [51]. Cells from which nuclear extracts were prepared were maintained as described below. After addition of the probe, the mixture was incubated for 10 min at room temperature before loading onto a 5% nondenaturing acrylamide gel containing 0.5 × TBE (1× Tris-Borate EDTA buffer is 0.09 M Tris-borate, 2 mM EDTA). Gels were electrophoresed for approximately 2 h, dried, and exposed to autoradiographic film for 1–2 d.

All cells were maintained in a water-jacketed incubator set to 37 °C with 5% carbon dioxide. The murine insulinoma βTC3 cells were grown in DMEM supplemented with 15% horse serum (Hyclone, Logan, Utah, United States), 2.5% fetal bovine serum (Hyclone), and penicillin/streptomycin. The neuro2A cells were maintained in MEM supplemented with 10% horse serum, 5% fetal bovine serum, 2 mM L-glutamine, 1% non-essential amino acids, and antibiotics. The T98G glioblastoma cells were grown in MEM containing Earle's Balanced Salt Solution (EBSS), 10% fetal bovine serum, 1% non-essential amino acids, sodium pyruvate, and antibiotics. The HepG2 cells and human embryonic kidney (HEK293) cells were maintained separately in MEM containing EBSS, 10% fetal bovine serum, 2mM L-glutamine, 1% non-essential amino acids, and antibiotics.

## Supporting Information

Figure S1 Haploview of the *GAD2* Region on Chromosome 10 This figure was generated using data from the International HapMap Project (http://www.hapmap.org/cgi-perl/gbrowse/gbrowse/hapmap) and using the program Haploview (http://www.broad.mit.edu/mpg/haploview/index.php). The SNPs studied are indicated on the diagram by the following numbers: rs2236418 (#2); rs3781118 (#3), rs3781117 (#4), rs1330581 (#12), and rs928197 (#34). On the diagram, the blue squares indicate missing data and unfilled red squares indicate a high degree of linkage disequilibrium (linkage disequilibrium coefficient, D′ = 1) between pairs of markers. Lesser degrees of linkage disequilibrium are indicated by the lighter red shading.
(95 KB PPT).

Figure S2 Haplotype Tag SNPs Required to Capture > 95% of the Haplotype Diversity within the *GAD2* Region The SNPs genotyped in the initial phase of the study (−243 A>G/rs2236418, +61450 C>A/rs992990, and +83897 T>A/rs928197) are indicated on the upper part of the diagram as markers 2, 24, and 35, respectively. Haplotypes are depicted as rows, with their population frequency shown at the right side of each row. The SNPs that are in complete linkage disequilibrium with each other are shaded the same color. In order to determine > 95% of the haplotype information within the *GAD2* region, genotypes at each of the SNPs indicated by the arrowheads (markers 1, 3, 4, 12, and 14, or a marker in perfect linkage disequilibrium with each) were required. To accomplish this, markers rs3781118 (#3 on diagram), rs3781117 (#4), and rs1330581 (#12) were genotyped in the second phase of the study.
(81 KB PPT).

Figure S3 Results from Transient Transfection of *GAD2* Reporter Genes in βTC3 Cells Containing the −243 A>G Polymorphism Three different sizes of luciferase reporter gene were constructed from the *GAD2* promoter (−1710/−4, −501/−4 and −1234/−4) for transfection into βTC3 murine insulinoma cells. Each WT reporter construct contains the −243 A allele, and the corresponding mutant reporter construct is identical to the WT except for the introduction of the −243 G allele. Twenty-four h before transfection, βTC3 cells were seeded in 6-well plates at a density of 250,000 cells/well containing DMEM supplemented with 10% fetal calf serum (Hyclone, Logan, Utah, United States), 2 mM L-glutamine, and penicillin/streptomycin. On the day of the experiment, each well was transfected with pGL3Basic or a *GAD2* promoter construct (0.4 μg) as well as 20 ng of the plasmid pRL-RSV (Promega), which encodes *Renilla* luciferase, to control for transfection efficiency. Transfections were performed in triplicate using Effectene reagent (Qiagen, Valencia, California, United States). Forty-eight h after transfection, cells were lysed, and firefly and *Renilla* luciferase assays were performed on the lysate using the Dual Luciferase Reporter Assay System (Promega), according to the manufacturer's standard protocol. Each experiment was repeated three times. We observed no significant difference in luciferase activity between each pair of WT and mutant *GAD2* promoter constructs.
(36 KB PPT).

Figure S4 EMSA Radiolabeled double-stranded oligonucleotide probes for each of the −243 A>G alleles were incubated with various nuclear extracts and electrophoresed in a 5% nondenaturing polyacrylamide gel. The arrow on the left side of the figure indicates the DNA-nuclear protein complex formed with βTC3 nuclear extract. Allele-specific differences in binding to βTC3 nuclear extract are seen in lanes 2 and 8.
(2.5 MB PPT).

Figure S5 Competitive EMSA Using Nuclear Extract from βTC3 Cells The complex formed by the interaction between the radiolabeled double-stranded probe containing the −243 A allele and βTC3 nuclear extract (indicated by the arrow) was competed away by the addition of excess amounts of unlabeled −243 A probe (lanes 3–6), but not by the addition of the same amount of unlabeled −243 G probe (lanes 7–10).
(101 KB PPT).

Table S1 PDT Results for rs3781117, rs3781118, and rs1330581 (32 KB DOC).

Table S2 Association Study Results for rs3781117, rs3781118, and rs1330581 in US and Canadian Groups None of the variant alleles at any of these three SNPs were associated with class III obesity in either of the two case-control groups or when pooled (for rs3781117, C allele: OR = 0.92, 95% CI 0.75–1.13, *p* = 0.43; for rs3781118, G allele: OR = 0.94, 95% CI 0.75–1.19, *p* = 0.62; for rs1330581, G allele: OR = 1.03, 95% CI 0.89–1.20, *p* = 0.65).
Can. Cases, Canadian cases.
(64 KB DOC).

Table S3 Genotype results for *GAD2* SNPs in US Caucasians, African Americans, and Africans Differences in allele frequency between ethnic groups were assessed by χ^2^ test. For the −243 A>G and +61450 C>A SNPs, the differences between Caucasian Americans (CA) and African Americans were highly significant (*p* < 0.001). For the −243 A>G SNP, the differences between CA, West Africans (WA), and North Africans (NA) were significant (CA vs. WA, *p* < 0.001; CA vs. NA, *p* = 0.014; WA vs. NA, *p* < 0.001). The frequency of the +83897 T>A SNP was also significantly different between CA and WA (*p* = 0.013). Other comparisons either yielded non-significant results, or were not conducted, as samples did not conform to Hardy-Weinberg equilibrium (indicated by an asterisk).
(53 KB DOC).

### Accession Number

The GenBank (http://www.ncbi.nlm.nih.gov/Genbank) accession number for *GAD2* is AY340073.

## Abbreviations

BMI: body mass index
bp: base pair
CI: confidence interval
EMSA: electrophoretic mobility shift assay
GABA: γ-aminobutyric acid
OR: odds ratio
PDT: pedigree disequilibrium test
SNP: single nucleotide polymorphism
WT: wild-type

## References

[pbio-0030315-b01] Calle EE, Thun MJ, Petrelli JM, Rodriguez C, Heath CW (1999). Body-mass index and mortality in a prospective cohort of U.S. adults. N Engl J Med.

[pbio-0030315-b02] Eckel RH, Krauss RM (1998). American Heart Association call to action: Obesity as a major risk factor for coronary heart disease. AHA Nutrition Committee. Circulation.

[pbio-0030315-b03] Kopelman PG (2000). Obesity as a medical problem. Nature.

[pbio-0030315-b04] Must A, Spadano J, Coakley EH, Field AE, Colditz G (1999). The disease burden associated with overweight and obesity. JAMA.

[pbio-0030315-b05] Stunkard AJ, Foch TT, Hrubec Z (1986). A twin study of human obesity. JAMA.

[pbio-0030315-b06] Maes HH, Neale MC, Eaves LJ (1997). Genetic and environmental factors in relative body weight and human adiposity. Behav Genet.

[pbio-0030315-b07] Barsh GS, Farooqi IS, O'Rahilly S (2000). Genetics of body-weight regulation. Nature.

[pbio-0030315-b08] O'Rahilly S, Farooqi IS, Yeo GS, Challis BG (2003). Minireview: Human obesity-lessons from monogenic disorders. Endocrinology.

[pbio-0030315-b09] Montague CT, Farooqi IS, Whitehead JP, Soos MA, Rau H (1997). Congenital leptin deficiency is associated with severe early-onset obesity in humans. Nature.

[pbio-0030315-b10] Strobel A, Issad T, Camoin L, Ozata M, Strosberg AD (1998). A leptin missense mutation associated with hypogonadism and morbid obesity. Nat Genet.

[pbio-0030315-b11] Clement K, Vaisse C, Lahlou N, Cabrol S, Pelloux V (1998). A mutation in the human leptin receptor gene causes obesity and pituitary dysfunction. Nature.

[pbio-0030315-b12] Jackson RS, Creemers JW, Ohagi S, Raffin-Sanson ML, Sanders L (1997). Obesity and impaired prohormone processing associated with mutations in the human prohormone convertase 1 gene. Nat Genet.

[pbio-0030315-b13] Krude H, Biebermann H, Luck W, Horn R, Brabant G (1998). Severe early-onset obesity, adrenal insufficiency and red hair pigmentation caused by POMC mutations in humans. Nat Genet.

[pbio-0030315-b14] Swarbrick MM, Vaisse C (2003). Emerging trends in the search for genetic variants predisposing to human obesity. Curr Opin Clin Nutr Metab Care.

[pbio-0030315-b15] Hager J, Dina C, Francke S, Dubois S, Houari M (1998). A genome-wide scan for human obesity genes reveals a major susceptibility locus on chromosome 10. Nat Genet.

[pbio-0030315-b16] Hinney A, Ziegler A, Oeffner F, Wedewardt C, Vogel M (2000). Independent confirmation of a major locus for obesity on chromosome 10. J Clin Endocrinol Metab.

[pbio-0030315-b17] Price RA, Li WD, Bernstein A, Crystal A, Golding EM (2001). A locus affecting obesity in human chromosome region 10p12. Diabetologia.

[pbio-0030315-b18] Boutin P, Dina C, Vasseur F, Dubois S, Corset L (2003). GAD2 on chromosome 10p12 is a candidate gene for human obesity. PLoS Biol.

[pbio-0030315-b19] Redden DT, Allison DB (2003). Nonreplication in genetic association studies of obesity and diabetes research. J Nutr.

[pbio-0030315-b20] Perusse L, Rankinen T, Zuberi A, Chagnon YC, Weisnagel SJ (2005). The human obesity gene map: The 2004 update. Obes Res.

[pbio-0030315-b21] Ioannidis JP, Ntzani EE, Trikalinos TA, Contopoulos-Ioannidis DG (2001). Replication validity of genetic association studies. Nat Genet.

[pbio-0030315-b22] Saar K, Geller F, Ruschendorf F, Reis A, Friedel S (2003). Genome scan for childhood and adolescent obesity in German families. Pediatrics.

[pbio-0030315-b23] Martin ER, Monks SA, Warren LL, Kaplan NL (2000). A test for linkage and association in general pedigrees: The pedigree disequilibrium test. Am J Hum Genet.

[pbio-0030315-b24] Hirschhorn JN, Lohmueller K, Byrne E, Hirschhorn K (2002). A comprehensive review of genetic association studies. Genet Med.

[pbio-0030315-b25] Barrett JC, Fry B, Maller J, Daly MJ (2005). Haploview: Analysis and visualization of LD and haplotype maps. Bioinformatics.

[pbio-0030315-b26] Cardon LR, Palmer LJ (2003). Population stratification and spurious allelic association. Lancet.

[pbio-0030315-b27] Editorial (1999). Freely associating. Nat Genet.

[pbio-0030315-b28] Cardon LR, Bell JI (2001). Association study designs for complex diseases. Nat Rev Genet.

[pbio-0030315-b29] Flegal KM, Carroll MD, Ogden CL, Johnson CL (2002). Prevalence and trends in obesity among US adults, 1999–2000. JAMA.

[pbio-0030315-b30] Hirschhorn JN, Altshuler D (2002). Once and again—Issues surrounding replication in genetic association studies. J Clin Endocrinol Metab.

[pbio-0030315-b31] Freedman DS, Khan LK, Serdula MK, Galuska DA, Dietz WH (2002). Trends and correlates of class 3 obesity in the United States from 1990 through 2000. JAMA.

[pbio-0030315-b32] Ovesjo ML, Gamstedt M, Collin M, Meister B (2001). GABAergic nature of hypothalamic leptin target neurones in the ventromedial arcuate nucleus. J Neuroendocrinol.

[pbio-0030315-b33] Cowley MA, Smart JL, Rubinstein M, Cerdan MG, Diano S (2001). Leptin activates anorexigenic POMC neurons through a neural network in the arcuate nucleus. Nature.

[pbio-0030315-b34] Pu S, Jain MR, Horvath TL, Diano S, Kalra PS (1999). Interactions between neuropeptide Y and gamma-aminobutyric acid in stimulation of feeding: A morphological and pharmacological analysis. Endocrinology.

[pbio-0030315-b35] Bannai M, Ichikawa M, Nishihara M, Takahashi M (1998). Effect of injection of antisense oligodeoxynucleotides of GAD isozymes into rat ventromedial hypothalamus on food intake and locomotor activity. Brain Res.

[pbio-0030315-b36] Kash SF, Johnson RS, Tecott LH, Noebels JL, Mayfield RD (1997). Epilepsy in mice deficient in the 65-kDa isoform of glutamic acid decarboxylase. Proc Natl Acad Sci U S A.

[pbio-0030315-b37] Schwartz MW, Sipols AJ, Grubin CE, Baskin DG (1993). Differential effect of fasting on hypothalamic expression of genes encoding neuropeptide Y, galanin, and glutamic acid decarboxylase. Brain Res Bull.

[pbio-0030315-b38] Leibowitz SF (1986). Brain monoamines and peptides: Role in the control of eating behavior. Fed Proc.

[pbio-0030315-b39] Rattan AK, Mangat HK (1990). Electrical activity and feeding correlates of intracranial hypothalamic injection of GABA, muscimol and picrotoxin in the rats. Acta Neurobiol Exp (Wars).

[pbio-0030315-b40] Noordmans AJ, Song DK, Noordmans CJ, Garrity-Moses M, During MJ (2004). Adeno-associated viral glutamate decarboxylase expression in the lateral nucleus of the rat hypothalamus reduces feeding behavior. Gene Ther.

[pbio-0030315-b41] Flier JS (2004). Obesity wars: Molecular progress confronts an expanding epidemic. Cell.

[pbio-0030315-b42] Saper CB, Chou TC, Elmquist JK (2002). The need to feed: Homeostatic and hedonic control of eating. Neuron.

[pbio-0030315-b43] Hinney A, Bornscheuer A, Depenbusch M, Mierke B, Tolle A (1997). Absence of leptin deficiency mutation in extremely obese German children and adolescents. Int J Obes Relat Metab Disord.

[pbio-0030315-b44] Pullinger CR, Hennessy LK, Chatterton JE, Liu W, Love JA (1995). Familial ligand-defective apolipoprotein B. Identification of a new mutation that decreases LDL receptor binding affinity. J Clin Invest.

[pbio-0030315-b45] Pullinger CR, Eng C, Salen G, Shefer S, Batta AK (2002). Human cholesterol 7alpha-hydroxylase (CYP7A1) deficiency has a hypercholesterolemic phenotype. J Clin Invest.

[pbio-0030315-b46] Ye S, Dhillon S, Ke X, Collins AR, Day IN (2001). An efficient procedure for genotyping single nucleotide polymorphisms. Nucleic Acids Res.

[pbio-0030315-b47] Chen X, Levine L, Kwok PY (1999). Fluorescence polarization in homogeneous nucleic acid analysis. Genome Res.

[pbio-0030315-b48] Spielman RS, Ewens WJ (1998). A sibship test for linkage in the presence of association: The sib transmission/disequilibrium test. Am J Hum Genet.

[pbio-0030315-b49] Dudbridge F (2003). Pedigree disequilibrium tests for multilocus haplotypes. Genet Epidemiol.

[pbio-0030315-b50] Purcell S, Cherny SS, Sham PC (2003). Genetic Power Calculator: Design of linkage and association genetic mapping studies of complex traits. Bioinformatics.

[pbio-0030315-b51] Schreiber E, Matthias P, Muller MM, Schaffner W (1989). Rapid detection of octamer binding proteins with ‘mini-extracts,' prepared from a small number of cells. Nucleic Acids Res.

