# Supplementary material for: Lack of Support for the Association between GAD2 Polymorphisms and Severe Human Obesity
Source: PLoS Biol. 2005 Aug 30;3(9):e315. doi: 10.1371/journal.pbio.0030315 (PMC1193520; doi:10.1371/journal.pbio.0030315)
Supplement: Supplementary file 1 [file pbio.0030315.st001.doc]

Supplemental Table 1. PDT results for rs3781117, rs3781118 and rs1330581.

| *SNP* | *N triads* | *Allele* | *Transmitted* | *Untransmitted* | *χ^2^ (df)* | *P* |
| --- | --- | --- | --- | --- | --- | --- |
| rs3781117 | 919 | T | 1610 | 1619 | 0.11 (1) | 0.75 |
|  |  | C | 232 | 223 |  |  |
| rs3781118 | 906 | T | 1665 | 1648 | 0.25 (1) | 0.61 |
|  |  | G | 151 | 168 |  |  |
| rs1330581 | 924 | T | 1379 | 1346 | 1.30 (1) | 0.25 |
|  |  | C | 469 | 502 |  |  |
