# Supplementary material for: Lack of Support for the Association between GAD2 Polymorphisms and Severe Human Obesity
Source: PLoS Biol. 2005 Aug 30;3(9):e315. doi: 10.1371/journal.pbio.0030315 (PMC1193520; doi:10.1371/journal.pbio.0030315)
Supplement: Supplementary file 2 [file pbio.0030315.st002.doc]

Supplemental Table 2. Association study results for rs3781117, rs3781118 and rs1330581 in U.S. and Canadian groups.

| *SNP* |  | *Genotype, N (frequency)* | | | *Allele frequency* | | *χ^2^*(df) | *P* |
| --- | --- | --- | --- | --- | --- | --- | --- | --- |
|  |  | TT | TC | CC | T | C |  |  |
| rs3781117 | U.S. Cases | 245 (0.81) | 50 (0.17) | 5 (0.02) | 0.900 | 0.100 | 2.66 (1) | 0.10 |
|  | U.S. Controls | 318 (0.76) | 93 (0.22) | 7 (0.02) | 0.872 | 0.128 |  |  |
|  | Can. Cases | 265 (0.74) | 89 (0.25) | 3 (0.01) | 0.867 | 0.133 | 0.19 (1) | 0.66 |
|  | Can. Controls | 555 (0.76) | 169 (0.23) | 8 (0.01) | 0.874 | 0.126 |  |  |
|  | **All cases** | **510 (0.78)** | **139 (0.21)** | **8 (0.01)** | **0.882** | **0.118** | **0.62 (1)** | **0.43** |
|  | **All controls** | **873 (0.76)** | **262 (0.23)** | **15(0.01)** | **0.873** | **0.127** |  |  |
|  |  |  |  |  |  |  |  |  |
|  |  | TT | TG | GG | T | G |  |  |
| rs3781118 | U.S. Cases | 236 (0.80) | 57 (0.19) | 3 (0.01) | 0.894 | 0.106 | 1.74 (1) | 0.19 |
|  | U.S. Controls | 350 (0.83) | 68 (0.16) | 2 (0.01) | 0.914 | 0.086 |  |  |
|  | Can. Cases | 309 (0.85) | 52 (0.14) | 2 (0.01) | 0.923 | 0.077 | 3.20 (1) | 0.074 |
|  | Can. Controls | 599 (0.81) | 133 (0.18) | 8 (0.01) | 0.899 | 0.101 |  |  |
|  | **All cases** | **545 (0.83)** | **109 (0.16)** | **5 (0.01)** | **0.910** | **0.090** | **0.25 (1)** | **0.62** |
|  | **All controls** | **949 (0.82)** | **201 (0.17)** | **10(0.01)** | **0.905** | **0.095** |  |  |
|  |  |  |  |  |  |  |  |  |
|  |  | AA | AG | GG | A | G |  |  |
| rs1330581 | U.S. Cases | 152 (0.51) | 114 (0.38) | 34(0.11) | 0.697 | 0.303 | 3.77 (1) | 0.052 |
|  | U.S. Controls | 227 (0.55) | 157 (0.38) | 27(0.07) | 0.743 | 0.257 |  |  |
|  | Can. Cases | 202 (0.54) | 139 (0.37) | 31(0.08) | 0.730 | 0.270 | 0.95 (1) | 0.33 |
|  | Can Controls | 382 (0.51) | 297 (0.40) | 68(0.09) | 0.710 | 0.290 |  |  |
|  | **All cases** | **354 (0.53)** | **253 (0.38)** | **65(0.10)** | **0.715** | **0.285** | **0.20 (1)** | **0.65** |
|  | **All controls** | **609 (0.53)** | **454 (0.39)** | **95(0.08)** | **0.722** | **0.278** |  |  |
