# Supplementary material for: Lack of Support for the Association between GAD2 Polymorphisms and Severe Human Obesity
Source: PLoS Biol. 2005 Aug 30;3(9):e315. doi: 10.1371/journal.pbio.0030315 (PMC1193520; doi:10.1371/journal.pbio.0030315)
Supplement: Supplementary file 3 [file pbio.0030315.st003.doc]

**Supplemental Table 3**. **Genotype results for *GAD2* SNPs in U.S. Caucasians, African-Americans, and Africans.**

| *SNP* | *Ethnic Group* | N | *Genotype, n (frequency)* | | | *Allele frequency* | |
| --- | --- | --- | --- | --- | --- | --- | --- |
|  |  |  | AA | AG | GG | A | G |
| -243A>G | Caucasian-American | 99 | 64 (0.65) | 29 (0.29) | 6 (0.06) | 0.79 | 0.21 |
|  | African-American | 98 | 9 (0.09) | 36 (0.37) | 53 (0.54) | 0.28 | 0.72 |
|  | West African | 55 | 2 (0.04) | 14 (0.25) | 39 (0.71) | 0.16 | 0.84 |
|  | North African | 36 | 14 (0.44) | 13 (0.41) | 5 (0.16) | 0.64 | 0.36 |
|  |  |  |  |  |  |  |  |
|  |  |  | CC | CA | AA | C | A |
| +61450C>A | Caucasian-American | 98 | 41 (0.42) | 47 (0.48) | 10 (0.10) | 0.66 | 0.34 |
|  | African-American | 99 | 23 (0.23) | 49 (0.49) | 27 (0.27) | 0.48 | 0.52 |
|  | West African* | 60 | 9 (0.15) | 43 (0.72) | 8 (0.13) | 0.64 | 0.36 |
|  | North African* | 34 | 6 (0.18) | 25 (0.73) | 3 (0.09) | 0.54 | 0.46 |
|  |  |  |  |  |  |  |  |
|  |  |  | TT | TA | AA | T | A |
| +83897T>A | Caucasian-American | 98 | 59 (0.60) | 35 (0.36) | 4 (0.04) | 0.78 | 0.22 |
|  | African-American | 98 | 60 (0.61) | 30 (0.31) | 8 (0.08) | 0.77 | 0.23 |
|  | West African | 59 | 25 (0.42) | 27 (0.46) | 7 (0.12) | 0.65 | 0.35 |
|  | North African* | 35 | 9 (0.26) | 24 (0.69) | 2 (0.06) | 0.60 | 0.40 |
